# Medial unicompartmental knee arthroplasty wear prediction using a total knee joint finite element model

**DOI:** 10.3389/fbioe.2025.1712322

**Published:** 2026-01-12

**Authors:** Xiaoyu Zhu, Zuming Mao, Rongkang Li, Shuqin Ma, Ruolan Lv, Feng Zhao

**Affiliations:** Key Laboratory of Biomechanics and Mechanobiology (Beihang University), Ministry of Education; Key Laboratory of Innovation and Transformation of Advanced Medical Devices, Ministry of Industry and Information Technology; National Medical Innovation Platform for Industry-Education Integration in Advanced Medical Devices (Interdiscipline of Medicine and Engineering); School of Biological Science and Medical Engineering, Beihang University, Beijing, China

**Keywords:** cartilage degeneration, finite element analysis, lateral compartment mechanics, polyethylene wear, unicompartmental knee arthroplasty

## Abstract

**Background:**

Unicompartmental knee arthroplasty (UKA) often fails due to osteoarthritis (OA) progression, insert wear, and other associated risks. Current wear studies on UKA focus on isolated prostheses, neglecting bones, cartilage and other knee joint structures. The aims of this study were to predict the wear of tibial inserts under simulated physiologically mechanical environment, investigate the influence of the presence of bone and cartilage tissue on wear simulation results, and explore the effect of wear on the mechanical environment of the lateral compartment.

**Methods:**

Two finite element models were developed: a UKA model consisting solely of prostheses, and a UKA total knee joint model (UKAK) incorporating prostheses, bones, cartilage, meniscus, and ligaments. Contact stress and wear prediction of medial inserts were analyzed under ISO standard loading. Furthermore, in the UKAK model, both wear prediction and the impact of wear on the lateral tibial cartilage were simultaneously examined.

**Results:**

The mass wear rates of UKA and UKAK model were 9.62 mg/million cycles and 7.41 mg/million cycles, respectively. The higher wear rate of the UKA model implied more evaluation testing requirements for the prosthesis. In contrast, the UKAK model, which better simulates physiological conditions, demonstrated that the maximum von Mises stress on the lateral tibial cartilage increased during the stance phase as gait cycles accumulated. After 5 million cycles, this stress increased by 27.53% at 43% of the stance phase compared to initial levels.

**Conclusion:**

Wear of the medial insert may increase lateral compartment cartilage stress, which may represent a potential mechanical risk factor associated with OA progression. This study provided support for the design optimization and clinical application of prostheses, and provided biomechanical data for the impact of wear on the mechanical environment of the lateral compartment.

## 1 Introduction

Unicompartmental knee arthroplasty (UKA) is an attractive alternative to total knee arthroplasty (TKA) for the treatment of end-stage unicompartmental osteoarthritis (OA) (Kugelman et al., 2025; Wu et al., 2022). Compared with TKA, UKA has advantages such as improved postoperative range of motion, earlier recovery of activity, shorter hospital stay, and fewer complications (Beard et al., 2020; Kugelman et al., 2025; Wu et al., 2022). However, the survival rate of the UKA is inferior to that of TKA (Hu et al., 2024).

Tibial insert wear is widely recognized as an important contributor to the failure of UKA (Makaram et al., 2024). Although registry data typically report polyethylene insert wear as the primary reason for revision in only a small proportion of cases (approximately 1%–2%), it serves as a critical underlying mechanism for a spectrum of late-term complications (AOANJRR, 2025; Dutch Arthroplasty Register, 2025; National Joint Registry, 2025). For instance, wear debris can induce periprosthetic osteolysis and aseptic loosening, and wear-related alterations in insert geometry and joint mechanics may contribute to altered loading patterns that may represent a potential mechanical risk factor for OA progression in the contralateral compartment (Migliorini et al., 2024; Purdue et al., 2006; Xie et al., 2023). Consequently, insert wear is closely associated with reduced long-term survival of UKA (Di Martino et al., 2021; Makaram et al., 2024; Huizinga et al., 2025). With rising demand among younger patients, improving UKA longevity is critical (Kurtz et al., 2009; Bernstein et al., 2024). Therefore, it is essential to study the wear of prosthetic inserts and their mechanical effects on the articular cartilage of the other compartment, in order to improve prosthetic design and extend the lifespan of UKA.

At present, there are two approaches to studying the wear of UKA: *in vitro* wear experiments and finite element analysis. *In vitro* wear simulation is a standard procedure for evaluating wear under different conditions during knee replacement surgery (Schwiesau et al., 2013). However, the current evaluation of UKA wear is based on parallel simulations of both medial and lateral UKA wear in the same dual compartment testing device, which differs from the mechanical environment of single compartment displacement *in-vivo* (Laurent et al., 2003; Grupp et al., 2009; Kretzer et al., 2011). Netter et al. (2015) and Koh et al. (2019a), Koh et al. (2019b), Koh et al. (2020b) used finite element methods to predict the effect of material and consistency of the prosthesis on wear. These wear-focused studies typically employed isolated UKA prosthesis modeling without incorporating bones and cartilage structures, thus unable to evaluate how progressive insert wear alters load transfer or stress distribution in the other compartment, significantly differing from *in vivo* conditions.

In parallel, numerous finite element studies have investigated the biomechanics of UKA and its effect on the contralateral compartment (Kang et al., 2018; Koh et al., 2020a; Ma et al., 2022). However, the implant geometry in these analyses is generally assumed to remain unchanged, and polyethylene wear is not considered. As a result, the potential feedback between progressive insert wear and the evolving biomechanical environment of the contralateral compartment has not been addressed. Exploring the effect of progressive UKA insert wear on stress redistribution in the contralateral compartment is crucial for improving our understanding of the mechanical factors potentially associated with OA progression after UKA.

In this study, two models were established: the UKA model containing only the prosthesis, and the UKA total knee joint (UKAK) model incorporating prostheses, bones, cartilage, meniscus, and ligaments. The purpose of this study is to investigate the influence of the presence or absence of bones and cartilage tissues on the simulation results of tibial insert wear and to explore the effect of wear on the mechanical environment of the lateral compartment. A hypothesis was proposed that the wear of the UKAK model might be reduced compared to the UKA model, and as wear aggravated, the cartilage stress in the lateral compartment would increase.

## 2 Methods

### 2.1 Establishment of the UKA model

**FIGURE 1:**
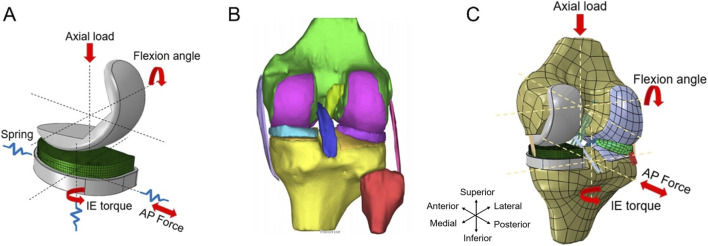
**(A)** The unicompartmental knee prosthesis (UKA) finite element model. **(B)** The 3D model of the intact knee joint. **(C)** The unicompartmental knee arthroplasty (UKAK) finite element model. AP, anteroposterior. I.E., internal-external.

The fixed bearing Persona® Partial Knee Prosthesis (Zimmer, Inc., Warsaw, IN, United States) was used in this study. The implant system consists of a femoral component, a tibial baseplate, and an 8-mm thick tibial insert (Figure 1A). Based on ISO 7207-1:2007 definitions, the femoral component measured approximately 20 mm in mediolateral (ML) width and 45 mm in anteroposterior (AP) length. The polyethylene insert measured approximately 26 mm (ML) × 41 mm (AP), while the tibial baseplate measured approximately 27 mm (ML) × 47 mm (AP). All dimensions were based on physical measurement of the components and rounded to the nearest millimeter for modeling clarity.

The UKA model was imported into Abaqus2021 (SIMULIA, Rhode Island, United States) for finite element analysis. Tetrahedron 10-noded elements C3D10M were used for femoral and tibial components. The tibial insert was meshed using hexahedron 8-noded elements C3D8R in Hypermesh2020 software (Altair Engineering, Inc., Troy, MI, United States). A mesh convergence analysis was performed for the UKA finite element model by monitoring the peak contact stress. The mesh was considered converged when the variation in peak contact stress between successive refinements was below 5% (Halloran et al., 2005). Based on this analysis, a global element size of 1.0 mm was selected for the final UKA model. The converged mesh comprised 181,243 nodes and 123,023 elements. The material of the tibia insert was conventional ultra-highmolecular-weight polyethylene (UHMWPE). The metal part was cobalt-chromium-molybdenum (CoCrMo) alloy. All parts of the UKA model were defined as linear-elastic isotropic materials and assigned values (Table 1) (Zhu et al., 2015; Zhang K. et al., 2019; Kwon et al., 2014).

**TABLE 1:** Material parameters of the main ligaments of the knee joint (Butler et al., 1990; Gardiner and Weiss, 2003; Peña et al., 2006).

Ligaments	C_10_ (MPa)	D_1_ (MPa^-1^)
ACL	1.95	0.00683
PCL	3.25	0.0041
MCL	1.44	0.00126
LCL	1.44	0.00126

Abbreviations: constants C_10_ and D_1_, neo-Hookean material coefficients; ACL, anterior cruciate ligament; PCL, posterior cruciate ligament; MCL, medial collateral ligament; LCL, lateral collateral ligament.

According to the ISO 14243 standard, gait loads were applied to the UKA model. The knee flexion angle and axial force were applied to the rotation center of the femoral component. Its ML and AP translations as well as varus–valgus (VV) and internal–external (I.E.,) rotations were constrained. The tibial control point was defined with reference to the geometric center of the inferior surface of the tibial baseplate, with a small inferior offset, and was kinematically coupled to the baseplate. The AP force and, I.E., torque were applied to the tibial control point. Its AP translation and, I.E., rotation were released, while axial and ML translations as well as flexion–extension (FE) and VV rotations were fixed.

Considering the influence of surrounding soft tissues on the movements of the knee joint, a torsion spring was applied to the tibial component for, I.E., rotation, with a stiffness coefficient of 0.6 Nm/deg (Koh et al., 2019a; Koh et al., 2019b). In addition, a pair of nonlinear springs were applied to the anterior and posterior directions of the tibial component, providing displacement constraints in the anterior and posterior directions of the tibia (Sathasivam and Walker, 1998). A penalty-based contact condition was specified at the tibial insert and femoral component interface with a friction coefficient of 0.04 (Koh et al., 2019a; Cui et al., 2024).

### 2.2 Establishment of the UKAK model

This study was approved by the board of research ethics (BM20230048), and informed consent was obtained from the participant. The right knee joint of a healthy adult man was selected for magnetic resonance imaging. In Mimics20.0 software (Materialise, Leuven, Belgium), the three-dimensional model of the knee joint was reconstructed (Figure 1B). The knee joint model included the femur, tibia, articular cartilage, meniscus, and the major ligaments of the knee joint: anterior cruciate ligament (ACL), posterior cruciate ligament (PCL), medial collateral ligament (MCL), and lateral collateral ligament (LCL). This intact knee joint finite element model has been validated in previous research (Yang et al., 2022).

The prosthesis was virtually implanted following the standard clinical surgical technique and the manufacturer’s guidelines for the Persona® Partial Knee System, under the guidance of an experienced orthopedic surgeon. Virtual bone resections were performed using Boolean operations to reproduce the clinical implantation procedure (Figure 1C). For the tibial component, the proximal tibial resection plane was defined perpendicular to the tibial mechanical axis in the coronal plane, with a posterior slope of 5° in the sagittal plane. No varus–valgus angulation or axial rotation was introduced. The tibial baseplate was positioned to maximize coverage of the resected medial tibial plateau. For the femoral component, the distal femoral resection plane was defined to restore the distal geometry of the medial femoral condyle and oriented approximately parallel to the tibial resection plane. The posterior femoral resection plane was aligned with the posterior condylar axis to define the anteroposterior position of the femoral component. The femoral component was positioned centrally on the medial femoral condyle, oriented perpendicular to the tibial component in the coronal plane, without axial rotation (Ma et al., 2022).

The UKAK model employed the same mesh convergence criterion as the UKA model and used an optimized multi-level mesh strategy. A fine element size of 1.0 mm was assigned to the implant components, 1.5 mm to the articular cartilage and meniscus, and 3.0 mm to the bony structures. The final UKAK model consisted of 322,881 nodes and 263,527 elements. Except for the prothesis, the element types of femoral cartilage, tibial cartilage, and meniscus were set to C3D10M, while the element types of the rest were set to C3D4.

The main ligaments of the knee joint were set as hyperelastic isotropic materials via the neo-Hookean model, and the element type was T3D2 (Weed et al., 2010; Yang et al., 2022). The material parameters of the ligaments were derived from experimental data (Butler et al., 1990; Gardiner and Weiss, 2003; Peña et al., 2006) and were listed in Table 1. Ligament-specific initial pre-strains were prescribed to represent the baseline physiological tension state of the intact knee joint, with values of 2% for the ACL, 0% for the PCL, 2% for the MCL, and 0.5% for the LCL. These values fall within reported physiological ranges and reflect the distinct functional roles of individual ligaments near the reference (near-extension) posture (Peña et al., 2006; Mesfar and Shirazi-Adl, 2005; Yang et al., 2010; Lahkar et al., 2021).The rest of the structures were defined as linear elastic isotropic materials and assigned parameters (Table 2).

**TABLE 2:** Material properties of different structures (Zhang K. et al., 2019; Kwon et al., 2014; Zhu et al., 2015).

Structures	Young’s modulus (MPa)	Poisson’s ratio
Bone	15,000	0.43
Meniscus	120	0.45
Cartilage	15	0.475
CoCrMo alloy	195,000	0.3
UHMWPE	940	0.46

Abbreviations: CoCrMo, cobalt-chromium-molybdenum; UHMWPE, ultra-highmolecular-weight polyethylene.

Simulated gait loads, according to the ISO 14243 standard, were applied to the UKAK model. The femoral loading point was defined as a point located 5.0 mm medial to the center of the femoral flexion–extension axis, consistent with the recommendations of ISO 14243. The flexion–extension axis was defined as the line passing through the geometric centers of curvature of the medial and lateral femoral condyles (Eckhoff et al., 2005). The knee flexion angle and axial load were applied at this femoral control point, without imposing any predefined load-sharing ratio between compartments. AP force and, I.E., rotation were applied to the center of the tibial plateau. The boundary conditions were defined as follows. For the tibia, AP translation and, I.E., rotation were released, while axial and ML translations as well as FE and VV rotations were constrained. For the femur, FE rotation was prescribed according to ISO 14243, axial translation and VV rotation were released, and all remaining degrees of freedom were fixed.

In addition, a user-defined VUAMP subroutine in ABAQUS was employed to represent passive soft-tissue restraints in the knee joint. The VUAMP subroutine defines the amplitude of applied forces and torques as a function of time and kinematic state. In this study, restoring AP forces and I.E. torques were generated only when the tibial AP displacement or, I.E., rotation exceeded predefined neutral zones, thereby simulating the passive constraint effect of surrounding soft tissues during gait (Yang et al., 2022). This implementation is consistent with the restraint concepts defined in ISO 14243.

### 2.3 Wear prediction of tibial inserts

The computational wear simulation utilized Archard wear Law (Archard, 1953) to calculate surface wear depth of insert:

$$H=K_{w}\sigma S$$

Where H is the wear depth, $K_{w}$ is an experimentally determined wear factor, σ is contact stress, and S is sliding distance. The wear factor employed in this study was 3.3 × 10^−7^ mm^3^/Nm (Saikko et al., 2001). Although obtained from standardized ball-on-flat testing, this wear coefficient has been widely used to represent the wear behavior of UHMWPE in knee arthroplasty simulations.

The wear algorithm used in this study was validated in previous finite element analysis and *in vitro* experiments conducted by Ding et al. (2018), and Mao et al. (2025). An adaptive remeshing procedure was introduced to simulate the surface wear progression. The adaptive wear simulation was carried out using Python scripts to interface with the Abaqus/Explicit output database (Knight et al., 2007). The simulation process was divided into 10 analysis steps, each representing 0.5 million cycles (MC) of wear, simulating a total of 5 MC of wear. For each iteration within an analysis step, the contact stress and sliding distance of the tibial insert surface nodes were extracted and input into Archard’s wear law. The wear depth for each surface node was calculated at the end of each iteration, and the surface nodes were then moved in a direction normal to the tibial insert surface. At the end of each analysis step, the tibial insert surface geometry was updated based on the total linear wear at each surface node. The mesh on the surface of the tibial insert was updated every 0.5 MC, which has been shown to produce results with a difference of only 2.75%–4.8% with a step size of 0.125 MC cycles (Knight et al., 2007; Wang et al., 2017). In order to compare the finite element results to the experimentally determined gravimetric mass loss, the wear volume was converted to a mass using a density of 0.93 mg/mm^3^ (Koh et al., 2019b).

The contact stress, contact area, wear rate, volumetric wear, and wear depth were calculated. In addition, to validate the wear model, the wear performance of the two models was compared with previously obtained results of experiments and finite element analysis.

## 3 Results

**FIGURE 2:**
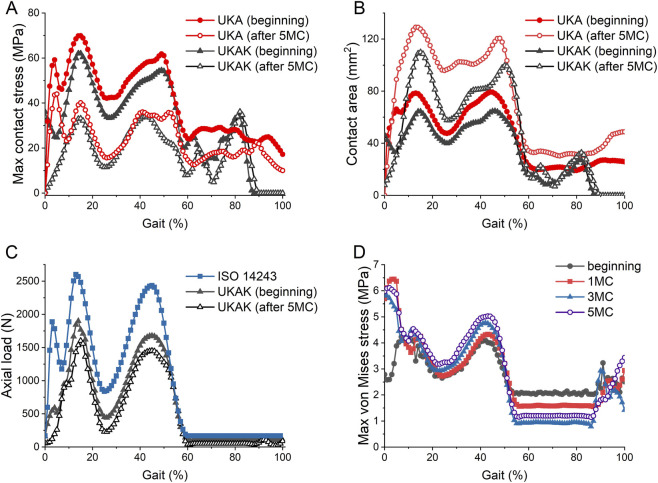
Comparison of the results at the beginning and after 5 million gait cycles. **(A)** The maximum contact stress on the tibial insert of the UKA and UKAK model. **(B)** The contact area on the tibial insert of the UKA and UKAK model. **(C)** Comparison of axial load in ISO 14243 standard with the axial load on the tibial insert of the UKAK model at the beginning and after 5 million gait cycles. **(D)** The maximum von Mises stress of the lateral tibial cartilage in the UKAK model.

The maximum contact stress and contact area of the insert in the UKA were greater than that of the UKAK (Figures 2A,B). After 5 MC, the maximum contact stress in both models decreased and the contact area increased compared to the beginning stage. Initially, the maximum contact stress of the insert during the gait cycle reached its maximum value at 14% of the cycle, which was 69.85 MPa in the UKA and 62.02 MPa in the UKAK. The maximum contact area in the UKA and UKAK was 80.72 mm^2^ and 68.02 mm^2^, respectively. After 5 MC, the maximum contact stress of the insert reached its maximum value at 15% of the cycle, which was 40.02 MPa in the UKA model and 33.16 MPa in the UKAK model. The maximum contact area in the UKA and UKAK was 132.09 mm^2^ and 115.84 mm^2^, respectively.

In the UKA, axial load was applied to the prosthesis according to the ISO 14243 standard. In the UKAK model, the axial load was applied to the knee joint and transmitted through both the medial and lateral compartments. At the first peak load of 2600 N (13% gait cycle), the medial tibial compartment bore approximately 71% of the total load, with a load of 1842.76 N on the medial side. After 5 million wear cycles, the medial load decreased to 1430.71 N, and its share of the total load reduced to approximately 59%. At the second peak load of 2,433.5 N (45% gait cycle), the medial load was 1677.57 N, or approximately 67%, with the medial load decreasing to 1450.93 N after 5 million cycles, reducing its share to around 60% (Figure 2C).

**FIGURE 3:**
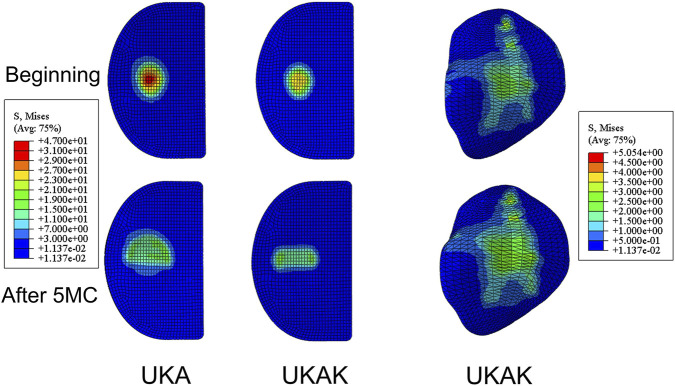
Von Mises stress distribution of insert in UKA model and von Mises stress distribution of insert and lateral tibial cartilage in UKAK model.

In the UKAK, after 5 MC, the maximum von Mises stress of the lateral tibial cartilage increased during the standing phase of the gait cycle and decreased during the swinging phase of the gait cycle compared to the beginning (Figure 2D). Initially, the maximum von Mises stress of the lateral tibial cartilage peaked at 42% of the gait cycle, which was 4.11 MPa. After 5 MC, it reached its peak value of 5.03 MPa at 43% of the gait cycle (Figure 3).

**FIGURE 4:**
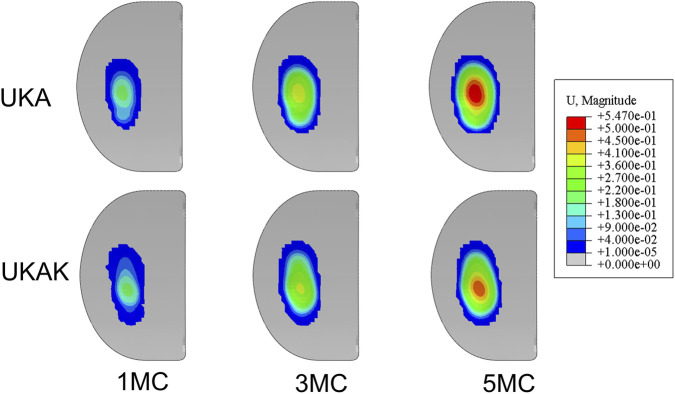
Predicted wear contour after 1, 3, 5 million cycles.

**FIGURE 5:**
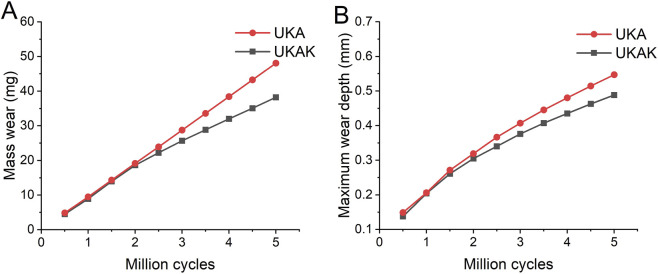
**(A)** Mass wear of UKA and UKAK models. **(B)** Maximum linear wear depth of UKA and UKAK models.

The computationally predicted wear contour of the tibial inserts in the UKA and UKAK under gait cycle loading was shown in Figure 4. The maximum linear wear depth and mass wear in the UKA were higher than those in the UKAK (Table 3). The mass wear rate of the UKA was 9.62 mg/MC, and that of the UKAK was 7.41 mg/MC. The mass loss between the two models began to show differences after 2 MC (Figure 5A). The growth rate of the mass loss and maximum linear wear depth in the UKAK slowed down as the number of gait cycles increased (Figure 5).

**TABLE 3:** Comparison of the gravimetric wear rate, gravimetric wear and wear depth.

Measurement	UKA	UKAK
Gravimetric wear rate (mg/MC)	9.62	7.41
Gravimetric wear (mg)	48.08	38.22
Wear depth (mm)	0.55	0.49

Abbreviations: UKA, a unicompartmental knee arthroplasty model consisting solely of prostheses; UKAK, a medial unicompartmental knee arthroplasty knee Joint model incorporating prostheses, bones, cartilage, meniscus, and ligaments; MC, million cycles.

## 4 Discussion

The novelty of this study lies in combining progressive medial UKA insert wear with a finite element model of the knee joint, enabling simultaneous prediction of polyethylene wear and evaluation of how wear-induced geometric changes influence the biomechanics of the contralateral compartment. In the UKAK model, as the number of gaits increased, the von Mises stress of the lateral tibial cartilage rose during the standing phase of the gait cycle, suggesting that wear of the medial insert may represent a potential mechanical risk factor for OA progression in the lateral compartment. The research hypothesis was confirmed. UKA model had a higher linear wear depth and mass wear rate than UKAK model. Under similar loading conditions, the model containing tissues such as bones and cartilage was closer to the *in-vivo* conditions of the knee joint, and should be fully considered in predicting insert wear.

**FIGURE 6:**
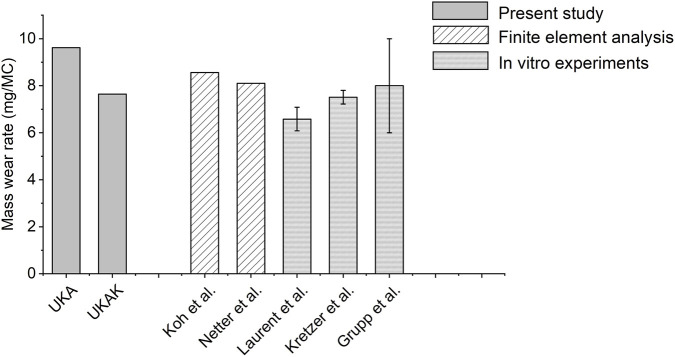
Comparison of mass wear rate between present study and other studies (Laurent et al., 2003; Koh et al., 2019b; Netter et al., 2015; Kretzer et al., 2011; Grupp et al., 2009).

The present models were validated compared with the *in vitro* experiment and finite element simulation results of previous studies (Figure 6) (Laurent et al., 2003; Koh et al., 2019b; Netter et al., 2015; Kretzer et al., 2011; Grupp et al., 2009). The location of the wear area is similar to the results in other literature, located in the middle and posterior of the insert. Although the wear predicted by the UKA model, which does not include structures such as bones and cartilage, is about 30% higher than the UKAK model, it is consistent with some previous research results. The prediction of excessive wear implied more evaluation testing requirements for the prosthesis.

The change curves of contact area and contact stress in the two models had two peaks, which are similar to the axial load input mode of ISO standards and basically consistent with the contact stress curve of Kretzer et al. (2011). These results are expected as axial loads dominate other loading forces such as AP or ML loads. Therefore, contact stress seems to be mainly affected by axial loads. Kwon et al. confirmed that the maximum contact pressure increases with the increase of axial force (Kwon et al., 2014). According to Archard’s law (Archard, 1953), contact pressure, contact area, and sliding distance are all important factors affecting wear. Research has shown that a significant reduction in surface contact stress helps to reduce wear (Zhang Q. D. et al., 2019; Koh et al., 2019b; Grupp et al., 2009).

The wear area of the UKA model was larger and deeper, but the wear positions of the two models were roughly the same. This difference likely stems from differences in load distribution: axial loads were directly applied to the prosthesis in the UKA model, whereas in the UKAK model, they were distributed across the entire joint with only part being borne by prosthesis. And as the number of gait cycles increases, wear causes a change in the geometric surface shape of the insert (Zhang Q. D. et al., 2019), leading to an increase in the conformity of the prosthesis. This change affects load distribution within knee joint and subsequently impacts peak stress on insert and lateral tibial cartilage. The results of this article are consistent with the conclusion of Fregly et al. (2010), that an increase in sagittal consistency of the prosthesis will decrease the predicted wear in a non-linear pattern, and as consistency increases, the decrease gradually decreases. The observed trend of medial load concentration leading to wear, which then triggers a compensatory lateral shift in load, is consistent with the expected mechanical behavior in knee prostheses. Future work will refine the model by incorporating more detailed soft-tissue properties and calibrating ligament pre-strain to achieve more physiologically accurate load distribution predictions.

As the number of gait cycles increases, the stress of the lateral tibial cartilage increased during the standing phase of the gait cycle. Numerous experimental, computational, and clinical studies have demonstrated that increased or abnormal stresses in articular cartilage are closely associated with cartilage degeneration and OA progression (Griffin and Guilak, 2005; Andriacchi and Mündermann, 2006; Bennell et al., 2011; Klets et al., 2018). The increased lateral compartment stresses, induced by wear in the medial compartment, may represent a potential mechanical risk factor for OA progression. Further research is needed to better understand how these mechanical factors affect cartilage health and lead to OA progression. Polyethylene wear and OA progression in the opposite compartment are the two important factors contributing to the failure of UKA implants (Kwon et al., 2014; Heaps et al., 2019). These two factors do not exist in isolation. The wear of the insert causes changes in the biomechanics of the lateral compartment, which may further affect the wear of the insert, leading to a vicious cycle of prosthesis wear and changes in lateral stress. Therefore, it is very necessary to explore the wear of the insert and the changes in the biomechanics of the lateral compartment in order to explore the mechanism of UKA failure and further optimize the design and clinical application of the prosthesis.

Several limitations of this study should be acknowledged. First, the UKAK model was developed based on a single-subject knee anatomy and a single implant design, which limits the generalizability of the results to other patient populations or implant configurations. Second, simplified material models were adopted for articular cartilage, menisci, and the polyethylene insert, which do not capture anisotropy, viscoelasticity, or time-dependent behavior and may affect absolute stress and wear magnitudes. Third, polyethylene wear was modeled using a classical Archard law with a single wear factor, without explicitly accounting for cross-shear, direction-dependent effects, creep, or thermal influences; therefore, the predicted wear results should be interpreted primarily in a comparative and trend-based manner rather than as precise quantitative values. Fourth, loading conditions were restricted to the ISO 14243 gait cycle, and further research is needed on different levels of activity. Finally, joint kinematics and medial–lateral load sharing are influenced by the selected degrees of freedom, ligament properties, and the VUAMP-based passive restraint formulation; although this approach follows established standards and represents physiologically meaningful constraints, variations in these parameters could affect load distribution and stress predictions. Future work will address these limitations by incorporating subject variability, advanced material and wear models, and dedicated *in-vitro* experimental validation.

## 5 Conclusion

This study presented a UKAK model incorporating prosthesis, bones, cartilage, meniscus and ligament. The wear depth and mass wear rate of this model were lower than that of UKA model. The UKAK model can better simulate physiologically mechanical environment and can predict the impact of wear on cartilage, which may further predict the vicious cycle of wear and cartilage degeneration in the future. Future wear studies should take into consideration the impact of bones, cartilage, and other tissues on wear mechanisms, in order to better improve prosthesis design and guide clinical applications.

## Data Availability

The raw data supporting the conclusions of this article will be made available by the authors, without undue reservation.
